# The Role of Chloride Channels in the Multidrug Resistance

**DOI:** 10.3390/membranes12010038

**Published:** 2021-12-28

**Authors:** Bartosz Wilczyński, Alicja Dąbrowska, Jolanta Saczko, Julita Kulbacka

**Affiliations:** 1 Faculty of Medicine, Wroclaw Medical University, L. Pasteura 1, 50-367 Wroclaw, Poland; bartosz.wilczynski@student.umed.wroc.pl (B.W.); alicja.dabrowska@student.umed.wroc.pl (A.D.); 2 Department of Molecular and Cellular Biology, Faculty of Pharmacy, Wroclaw Medical University, Borowska 211A, 50-556 Wroclaw, Poland; jolanta.saczko@umw.edu.pl

**Keywords:** multidrug resistance, chloride channels, volume regulated channels

## Abstract

Nowadays, one of medicine’s main and most challenging aims is finding effective ways to treat cancer. Unfortunately, although there are numerous anti-cancerous drugs, such as cisplatin, more and more cancerous cells create drug resistance. Thus, it is equally important to find new medicines and research the drug resistance phenomenon and possibilities to avoid this mechanism. Ion channels, including chloride channels, play an important role in the drug resistance phenomenon. Our article focuses on the chloride channels, especially the volume-regulated channels (VRAC) and CLC chloride channels family. VRAC induces multidrug resistance (MDR) by causing apoptosis connected with apoptotic volume decrease (AVD) and VRAC are responsible for the transport of anti-cancerous drugs such as cisplatin. VRACs are a group of heterogenic complexes made from leucine-rich repetition with 8A (LRRC8A) and a subunit LRRC8B-E responsible for the properties. There are probably other subunits, which can create those channels, for example, TTYH1 and TTYH2. It is also known that the ClC family is involved in creating MDR in mainly two mechanisms—by changing the cell metabolism or acidification of the cell. The most researched chloride channel from this family is the CLC-3 channel. However, other channels are playing an important role in inducing MDR as well. In this paper, we review the role of chloride channels in MDR and establish the role of the channels in the MDR phenomenon.

## 1. Introduction

Cancer cases have significantly increased over the last decade. The mainstay of treating these diseases is a surgical procedure, followed by radiotherapy and chemotherapy involving cytostatic drugs [1]. Unfortunately, the clinical effects of cytostatic drugs are weakened by drug resistance in some cancer cells. Therefore, new, unconventional methods of cancer treatment are needed. One of the most effective anti-cancer drugs is preparations based on platinum-containing compounds, such as cisplatin, carboplatin, or oxaliplatin, but their most significant disadvantage is toxicity and resistance developing during therapy. It is also essential to understand the mechanisms responsible for the drug resistance process of cancer cells. This would allow for better clinical effects in cancer treatment by implementing new procedures to prevent drug resistance.

There may be distinguished intrinsic (i.e., present before exposure to chemotherapy) and acquired drug resistance. Anticancer drugs (cytotoxic, immunotherapeutic, and hormonal drugs) are usually active at the beginning of treatment, after which resistance to therapy appears [2]. Recurrent cancer cells show increased resistance to chemotherapeutic drugs [3], which significantly reduces the effectiveness of the treatment method [4]. Several mechanisms influencing tumor cell resistance have already been identified. It has been shown that membrane transporters and ion channels play a key role in chemosensitivity [5]. In the last decade, intensive research has been carried out on the molecular mechanisms linking ion channels’ expression and/or function with resistance to chemotherapy [6]. The ion channels connected with multidrug resistance include chloride channels, which are the structures we will focus on most.

One of the best and well-researched chloride channels is CFTR, which enables cAMP-stimulated anion conductance. It is well-known that if this channel is defective, cystic fibrosis (CF) occurs [7]. However, questions whether other chloride channels play an important role in any pathological mechanisms arose. It turns out that chloride channels are not only overexpressed in numerous tumors, but, also, they might induce drug resistance phenomenon. Chloride channels might be divided into the following groups: CFTR; volume regulated anion channels (VRAC), calcium-activated chloride channels, Maxi-chloride channels, and the CLIC (or ClC) family consisting of nine known channels [6]. However, mainly channels from the VRAC and ClC families are involved in those mechanisms. There is evidence that ClC family is involved in creating MDR in mainly two mechanisms—by acidifying the cell and changing the cell’s metabolism. Although the most well-known chloride channel in this phenomenon is ClC-3, there is also proof that other members, such as ClC-1 or ClC-5, induce resistance at some point [8,9,10]. This paper focuses on describing and comparing possible ways of creating drug resistance, and thus finding possible therapeutic targets.

## 2. VRAC

### 2.1. The Basic Physiological Role of VRAC

**Figure 1:**
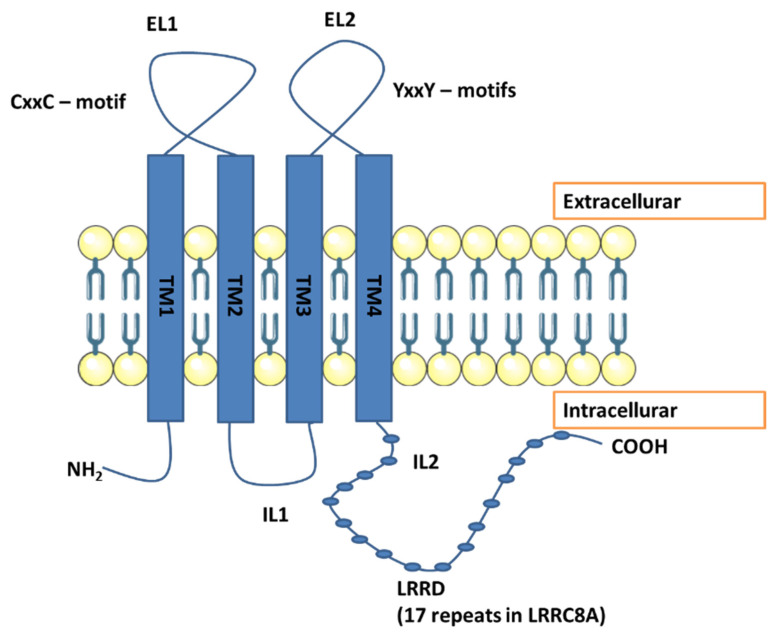
The figure shows the structure of the LRRC8 subunits. High-resolution cryo-electron microscopy enabled to visualization of the structure of LRRC8 subunits 145,146. Studies have shown the presence of 4 permeable transmembrane helices (TM) located at the amino terminus and the carboxyl terminus, which contain 17 leucine-rich repeats. Moreover, the TM1-TM2 (EL1) and TM3-TM4 (EL2) domains are extracellularly located, and the amino and carboxyl terminus and the TM2-TM3 (IL1) and IL2 domain to be located intracellularly. According to the study of the LRRC8A protein sequence and knowledge of the domains interacting with cisplatin, the existence of cisplatin-interacting domains within LRRC8A, such as methionine in cellular and transmembrane segments, and metal-binding CxxC/YxxY motifs (letters denote specific amino acids, x is a random amino acid) [27].

Although the osmolarity of the extracellular fluid is about 300 mOsm, the body’s cells are constantly exposed to changes in their volume caused by electrolytes and water flowing through the cell membrane [11]. Fortunately, cells can regulate their volume, thanks to which it is possible to maintain cell homeostasis and adapt to many different physiological processes, such as migration and proliferation [12,13]. This ability is conditioned by the numerous transport proteins found in the plasma membrane. They allow the flow of inorganic ions and larger organic osmolytes from and into the cell, which then causes an osmotic flow of water [14]. These include numerous anionic and cationic channels, such as the volume regulated anion channel (VRAC), also known for its low outward straightening currents by the volume sensitive outward righting channel (VSOR). This channel is activated in response to an increase in the cell volume and conditions the outflow of chlorides and organic osmolytes from the cell, thus leading to a regulatory volume decrease (RVD) [14,15,16,17,18]. The relative sequence of anion permeability characteristic for this channel is characterized by the level of I→NO_3_→Br→Cl→F [19]. In addition, these channels are conducted by larger osmolytes such as taurine and myo-inositol, for which these channels are referred to as volume-sensitive organic osmolyte/anion channels (VSOAC) [20,21,22,23]. Discrepancies between channel time course for swelling induced activation/inactivation, osmotic set-point, sensitivity to cholesterol depletion, and sensitivity to membrane potential have led to the hypothesis that VRAC and VSOAC are separate entities [24,25]. It is now believed that the volume-sensitive VRAC and VSOAC transporters are heteromers composed of subunits derived from the LRRC8 family (LRRC8A…E), which determines the specificity and kinetics of channel activation [16,26]. Moreover, recently, it was possible to visualize the structure of these subunits (Figure 1).

### 2.2. The Role of VRAC in Migration, Proliferation, and Apoptosis

Proliferation and migration are among the basic physiological processes in which VRAC is involved [13,28]. During the cell cycle, after the initial increase in volume, Cl^−^ channels’ transient activation leads to a decrease in volume [29]. The use of various non-specific VRAC inhibitors led to a decrease in proliferation rate in various types of cells [30,31,32,33,34,35]. After using VRAC inhibitors, lung cancer cells, cervical cancer cells, and T lymphoma cells were halted in the G0/G1 phase, which may mean that VRAC is necessary to pass the G1/S transition phase [36,37]. Similarly, VRAC is associated with the proliferation of nasopharyngeal cancer cells and human ovarian cancer cells, and VRAC inhibitors inhibited the transition from the G1 to S phase of these cells [36]. In addition, suppression of LRRC8A inhibited the growth of colon and hepatocellular carcinoma cells in vivo in nude mice [38,39]. Cell migration is associated with the differential activity of ion channels and transporters involved in local changes in cell volume, including VRAC. During this process, the volume increases in the front part of the migrating cell, and the volume decreases in its posterior part [28]. VRAC inactivation with VRAC inhibitors and a hypertonic solution reduced the mobility of nasopharyngeal cancer cells and glioblastoma cells [40,41], and glycine-induced cell swelling was correlated with microglial cell migration [42]. It has also been shown that DCPIB, a known VRAC inhibitor, inhibits the migration of glioblastoma cells [35], and that siRNA treatment directed against LRRC8A led to inhibition of migration of human colon cancer cells HCT116 [39].

**Figure 2:**
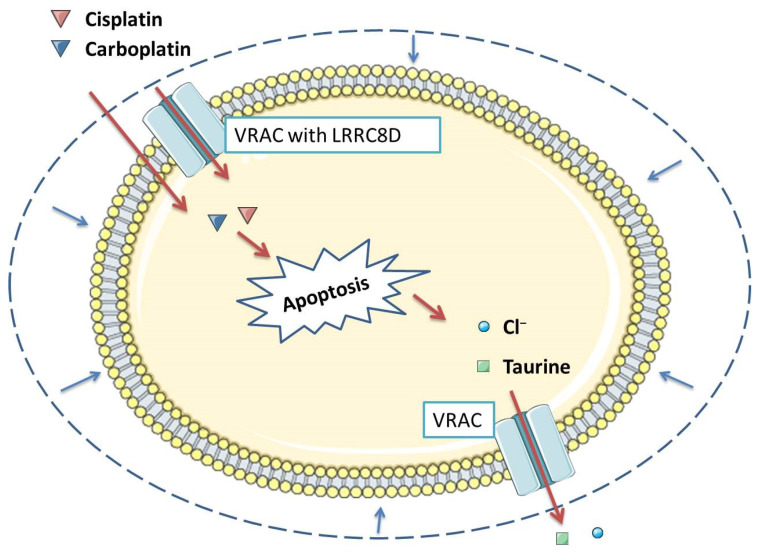
Volume-regulated anion channel (VRAC) is involved in multi drug resistance in two ways. On the one hand, through VRAC containing the LRRC8D subunit, platinum-based anti-cancer chemicals such as cisplatin and carboplatin enter the cell, which then induces apoptosis. On the other hand, VRAC, through the release of chlorides and organic osmolytes from the cell, participates in apoptosis volume decrease (AVD), which is associated with the occurrence of apoptosis in many types of tumor cells [43,47,48].

The third important process related to the functioning of VRAC is apoptosis [43,44], which is the process of programmed cell death that ensures a homeostatic balance between the rate of cell formation and cell death [45]. An important standpoint of this phenomenon is the flow of intracellular ions, which occurs both in the executive and initial phases of apoptosis [46]. This flow is responsible for the cell contraction at the onset of apoptosis, defined as the reduction in the volume of apoptosis (AVD), in which VRAC is involved, analogous to RVD [47,48] (Figure 2). When examining the Ehrlich tumor cells responsible for wild-type (WT) and multi-drug resistant MDR ascites (EATC), AVD was divided into three stages: early AVD1 lasting 4 to 12 h, transient AVDT lasting 12 to 32 h, and secondary AVD2 lasting 32 h. AVD1 and AVD2 were associated with a net loss of Cl^−^, K^+^, Na^+^, and amino acids leading to water loss by the cell. In AVDT, Na^+^ and Cl^−^ were accumulated and cell volume was partially recovered. MDR EATC compared to WT underwent less pronounced AVD1, increased AVDT, and delay in AVD2 induction, which led to reduced loss and increased Cl^−^ uptake [49]. However, the role of AVD in apoptosis is controversial. Apoptosis of many cells requires a reduction in their volume [50,51,52]; however, there are situations in which AVD is not required for apoptosis to occur [53,54]. It now appears that AVD does not have to be strictly required for programmed cell death, but is a process often associated with apoptosis [47,55,56]. An in vitro Fas ligand-induced vascular atrophy and apoptosis model in smooth muscle cells showed increased expression of LRRC8A21. It has also been observed that inducers of apoptosis, such as staurosporine, activate VRAC independently of cell swelling [28,57,58]. Moreover, cisplatin, which is a known anti-cancer drug responsible for cell apoptosis, activated LRRC8 [26,59,60].

### 2.3. Pharmacological Inhibition of VRAC Impairs Apoptosis Induced by Various Compounds, Including Cisplatin

It was found that 4-acetamido-4′-isothiocyanostilbene-2,2′-disulfonic acid (SITS), a stilbene derivative, which is a known blocker of both Cl^−^/HCO3- exchangers and Cl^−^ channels, induces, independently of intracellular pH, cisplatin resistance in murine breast tumor cells [61] and canine osteosarcoma cells [62]. Another stilbene derivative, 4-4′-diisothiocyanostilbene-2,2′-disulfonic acid (DIDS), to which VSORs are sensitive was also found to inhibit apoptosis, which is induced in human epidermal cancer KB cells, mediated by cisplatin and reduces the sensitivity of Kb cells to 5-day exposure to cisplatin [63]. Moreover, the nonstilbene derivative floretin, a VSOR blocker, inhibits cisplatin-induced caspase-3 activation in KB cells, thereby inhibiting apoptosis [59]. In human HeLa epithelial cells, human lymphoid U937 and murine neuroblastoma × rat glioma NG108-15 and rat PC12 pheochromocytoma, apoptosis was initiated by the use of staurosporine (STS) a known mitochondrial apoptosis inducer or inducer-mediated inducer of apoptosis, tumor necrosis factor α plus cycloheximide (TNF/CHX) [15,64,65]. Early-phase cell contraction associated with apoptosis, termed AVD, and all apoptotic events were completely inhibited by the C1 channel blocker, 5-nitro-2-(3-phenylpropylamino) benzoate (NPPB) or DIDS, SITS, niflumic acid, and glibenclamide49, which are known blockers of volume-sensitive Cl^−^ channels61. In contrast, blockers of cAMP-activated Cl^−^ channels (CFTR) and Ca^2+^-activated epithelial Cl^−^ channels remained ineffective [15]. These results suggest that volume-regulating Cl^−^ channels are involved in AVD. Other studies showed that DIDS showed an inhibitory effect on STS-induced DNA fragmentation monitored by dUTP terminal labeling in rat cerebellar granular neurons at much higher concentrations. The use of Ba^2+^ and quinine, known blockers regulating the volume of K^+^ channels, also prevented all apoptotic events analyzed in this study [15]. Other K^+^ channel blockers, TEA or 4-aminopyridine, blocked apoptotic cell death in other cell types [66,67,68]. Thus, it appears that K^+^ channel activity is involved in AVD in conjunction with Cl^−^ channel activity. The use of the NS3728 anion channel inhibitor led to the inhibition of AVD and the abolition of differences in AVD, ion movement, and caspase 3 activations between Ehrlich tumor cells causing wild-type (WT) and multi-drug resistant (MDR) (EATC) ascites. NS3728 also inhibited the loss of NPS that is induced by cisplatin after 3–8 h, while taurine release associated with volume regulation is strongly reduced in the MDR of the EATC, suggesting that inhibition of NS3728-sensitive amino-permeable channels may also be involved in preventing apoptosis [49]. After using VSOR blockers, floretin or NPPB, STS-induced AVD was inhibited in HeLa cells, followed by suppression of caspase 3 and 7 activations, responsible for transmitting signals from the cell membrane to the nucleus in response to several stimuli, including stress, participate in apoptotic cell death [69]. VSOR blockers reduced the induction of AVD and the phosphorylation of stress-responsive MAPKs induced by STS, but suppression of these MAPKs did not hinder AVD [70]. This suggested that sustained cell contraction or AVD is an independent and preceding event in the MAP kinase cascade. It was also later shown that the use of the chloride channel blockers NPPB and tamoxifen slowed the apoptosis rate and inhibited the proliferation of MG 63 osteosarcoma cells initially induced by cisplatin [57]. Exposing murine MCE301 colon epithelial cells to 4-(2-butyl-6,7-dichloro-2-cyclopentylindan-1-one-5-yl) oxybutyric acid (DCPIB), a specific VSOR blocker [71], the early apoptosis induced by sodium butyrate was inhibited [71], which induces apoptotic cell death in various cell types, including colon epithelial cells [72,73].

### 2.4. Various Cisplatin-Resistant Tumor Cell Lines Show Reduced VSOR Currents

Model cells characterized by cisplatin resistance are KCP-4 cells derived from the human epidermal cancer cell line KB-3-173. KCP-4 cells, unlike parental KB-3-1 cells (referred to as KB cells), will not undergo apoptosis when grown in a medium containing cisplatin at a concentration of approximately 23 µM. It was also found that KCP-4 cells had a reduced cisplatin accumulation compared to the KB parentage. An active volume-sensitive, outward straightening channel [74] Cl^−^ (VSOR) was found in the KB cell line; moreover, the Cl^−^ currents increased after cisplatin treatment, which was associated with increased cisplatin-induced apoptosis [51]. KCP-4 cells, on the other hand, were characterized by practically absent functional VSOR expression [75]. It would be expected that once the VSOR channels were restored in KCP-4 cells, they would be more susceptible to cisplatin-induced apoptosis. Indeed, after treatment with trichostatin A (TSA), a histone deacetylase inhibitor that partially restored VSOR chloride channel function, KCP-4 cells treated with cisplatin showed an increase in caspase-3 activity after 24 h and a decrease in cell viability after 48 h [75]. Moreover, after treating these cells with the VRAC channel inhibitor, their initial resistance to cisplatin-induced apoptosis was restored [75]. A few years later, a similar study was carried out using cisplatin-resistant A549/CDDP cells that arose from lung adenoma cells or wild-type A549 cells treated with gradually increasing doses of cisplatin. A549/CDDP cells did not show Cl^−^ and AVD currents after treatment with cisplatin, unlike A549 cells, which, when stimulated with cisplatin reverse, showed currents such as AVD, and underwent apoptosis. Upon applying the chloride channel blocker DIDS on A549 cells, they ceased to show AVD and Cl^−^ currents. On the other hand, A549/CDDP cells, like KCP-4 cells, after treatment with trichostatin A and its recovery of VSOR Cl_2_ activity, showed reduced resistance to cisplatin [76]. The TSA did not fully restore the VSOR Cl^−^ currents, and the mechanism behind it is not fully known [75]. It is known that TSH influences gene expression by inhibiting histone deacetylase (HDAC), which, together with histone acetyltransferases, affects chromatin transcription by regulating the level of histone acetylation [77,78]. Inhibition of HDAC causes chromatin to adopt a more open configuration, which will condition the activation of silenced genes [79]. This means that TSA may increase the expression of the gene encoding the VSOR Cl^−^ channel, change the expression of the channel regulator gene or affect the expression of many genes that affect the activity of the VSOR Cl^−^ channel [75].

### 2.5. Low Expression of LRRC8A Is Associated with Increased Resistance to Clinically Relevant Levels of Cisplatin

The available studies indicated, that in cisplatin sensitive human ovarian carcinoma cells (wild-type (WT)), cisplatin-induced osmolyte loss correlates with increased LRRC8A expression, while cisplatin resistance in A2780 RES cells correlates with decreased LRRC8A expression. It was found that the 18-h cisplatin exposure resulted in a 2- to 2.5-fold increase in the LRRC8A protein content in A2780WT, while its expression in A2780CisR cisplatin-resistant cells remained unchanged [80]. Accordingly, altering LRRC8A activity may be used as a biomarker for the progression of apoptosis and the acquisition of drug resistance. LRRC8A MRNA is also reduced in RES A2780 cells compared to WT A2780 cells, although not to the same extent as LRRC8A protein expression [80]. This indicates that post-translational ubiquitination and degradation of LRRC8A may be increased during the acquisition of cisplatin resistance in A2780 cells, as indicated by Bradley et al. [81]. In addition, during the development of cisplatin resistance in A2780 cells, taurine accumulates in them as a result of, among other things, a decrease in VSOAC expression. In the context of apoptosis, supplementation with this amino acid reduces programmed cell death induced by drug action, hypoxia, and ischemia through mechanisms including counteracting changes in the permeability of mitochondrial membranes and limiting cytochrome c release [82], preventing Bax and Fas upregulation [83], p53 activity limitation [84], ROS and Ca^2+^ mobilization [85,86], increasing the amount of cellular Bcl-2 [87], inhibiting the suppression of the apoptosome Apaf-1 complex [88] and reducing the expression of caspase-8 and caspase-9 [26].

**Figure 3:**
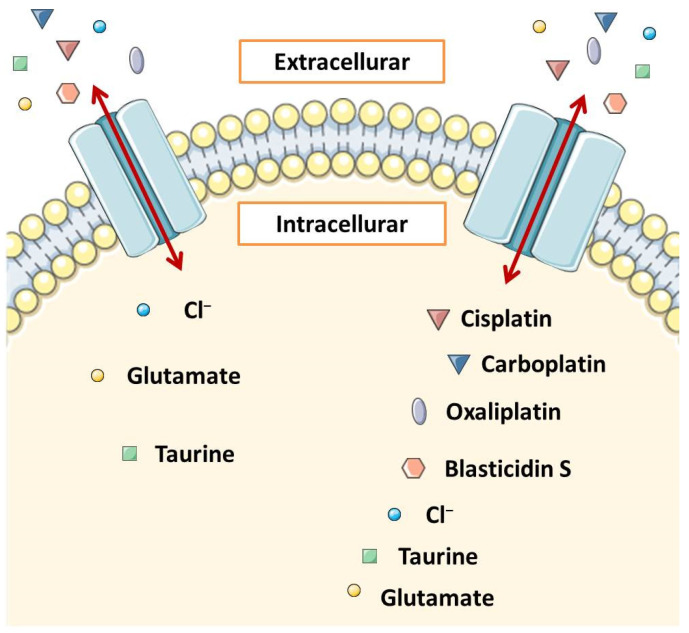
The schematic representation of the volume-regulated anion channel (VRAC). VRACs are heteromers composed of subunits belonging to the LRRC8 family. The main component is the leucine-rich 8A repeat that forms the LRRC8A. This subunit is accompanied by four homologous subunits that belong to the group LRRC8B-LRRC8E, and the channel properties depend on the type of these subunits. The connection of LRRC8A with LRRC8D (right-hand channel) is particularly important. It determines the formation of a VRAC with a relatively large diameter, allowing larger molecules such as cisplatin, blasticidin S, and oxaliplatin to pass through this channel into the cell interior. VRACs that lack the LRRC8D subunit (left channel) have a smaller diameter and transport smaller molecules. LRRC8A/C and LRRC8A/E pairs transport cisplatin only in a marginal manner [26,93].

A landmark paper on the role of VRAC in resistance to platinum-based cancer therapy was published in 2015. To date, resistance associated with reduced VRAC expression has been associated primarily with impairment of AVD. However, it turned out that heteromeric LRRC8 channels (VRAC) may be involved in the transport of cisplatin, carboplatin, and other widely used anticancer drugs into the cell and that the substrate selectivity and pharmacology of VRAC are related to the subunits that build them [26]. It has already been shown that the hypotonic swelling of the cell stimulated the cell to take up cisplatin [11]. This uptake was eliminated in cells with LRRC8A but no LRRC8 B, C, D, or E, and in cells with LRRC8B, C, D, and E but no LRRC8A cells that did not have functional VRAC and were inhibited by carbenoxolone, which is a VRAC inhibitor [26]. Approximately 50 to 70% of the long-term isotonic cisplatin uptake was mediated by the LRRC8D and LRRC8A heteromers, but only marginally by the LRRC8A/C and LRRC8A/E pairs [26] (Figure 3). VRAC most likely takes place by passive diffusion through the plasma membrane [89]. The highest possible VRAC radius is 0.63 nm [90], while the mean radii of cisplatin and carboplatin are 0.30 nm and 0.40 nm, respectively [91]. It means that VRAC can transport these compounds. Previously, VSOAC was also considered to be a potential channel involved in cellular transport and uptake of larger compounds such as the antibiotic blasticidin S58 and the chemotherapeutic drug daunorubicin [92]. VRAC pores carrying LRRC8D can carry blasticidin S and oxaliplatin [93] (Figure 3). Another group concluded that cisplatin uptake in human ovarian cancer cells (A2780) depends on the presence of the LRRC8A subunit and is sensitive to volume changes and that cisplatin resistance of these cells is associated with decreased LRRC8A expression. The content of Pt in a cell is high when VSOAC channels are activated as a result of hypoosmotic cell swelling or membrane depolarization, and it is reduced when LRRC8A expression is reduced due to gene extinction, pharmacological inhibition of channels, or cells have acquired a phenotype characterized by low LRRC8A expression. Moreover, based on information about domains interacting with cisplatin in other transporters such as CTR1 or ATP7A/B and the analysis of the LRRC8A protein sequence, it has been proposed that LRRC8A contains domains that can attract cisplatin, e.g., methionine in cell and transmembrane segments and CxxC/YxxY [27] (Figure 1). Further study of this group confirmed that downregulation and decreased activation of LRRC8A correlate with the development of resistance to cisplatin-induced apoptosis in A2780 cells and showed that it correlates with resistance in human alveolar cancer cells (A549) [78]. The LRRC8A correlation was also investigated with the human p53 protein [78], often referred to as the “guardian of the genome” for its ability to control apoptosis, DNA repair, autophagy, and proliferation by influencing the expression of various genes and miRNAs96. Treatment with cisplatin leads to an increase in the stability of the p53 protein in the cell due to ATR/ATM-mediated phosphorylation [87], which further results in the transcription of the corresponding proteins, ultimately leading to inhibition of cell proliferation or the initiation of apoptosis [94]. Presumably, LRRC8A forming VSOR/VSOAC is required for an increase in the level of p53 protein and an increase in the level of its downstream signaling elements, i.e., MDM2 and p21Waf1/Cip1 expression, and cisplatin-induced caspase-9 and -3 activation in A2780 human ovarian cancer cells, such as and in A549 alveolar carcinoma cells, as pharmacological inhibition and transient knockdown of LRRC8A inhibited these processes [78]. In contrast, pharmacological inhibition of anion channels had little effect on the initiation of caspase-3-related apoptosis by TNFα and hyperosmotic cell contraction [78].

### 2.6. Unknown VRAC Subunits Other Than LRRC8

It appears that cisplatin-resistant cells with decreased VRAC activity do not always show decreased expression of LRRC8 subunits relative to the parental cell lines that are sensitive to cisplatin [95,96]. LRRC8A mRNA is expressed similarly in both KB cells belonging to the cis platinum-sensitive cell line, which is rich in VSOR, and KCP-4 cells, which belong to the human cisplatin-resistant cell line and are characterized by a VSOR deficiency. Moreover, the expression level of the LRRC8A protein itself was similar in KCP-4 cells and VRAC-rich cells [95]. Even though LRRC8A is an essential element of VRAC, its overexpression reduces the native VRAC currents in VRAC-rich cells [22,23,58,97] and, also, reduces the already small native VRAC currents in KCP-cells [95]. This effect may be related to the concentration dependence of transfected LRRC8A, which is U-shaped in the graph; furthermore, this unexpected effect disappears when LRRC8D or LRRC8E are expressed simultaneously with LRRC8A. Moreover, LRRC8D and LRRC8E mRNA levels were similar in KCP-4 cells and KB cells and comparable to that in HEK293T, HeLa cells and 407 intestines, which are rich in VRAC. There was also no association between LRRC8C and LRRC8E knockdown and VSOR inactivation in HeLa cells [95], in contrast to the results obtained with HCT116 [98,99]. It is possible, then, that the molecular accelerator and slower associated with the VRAC inactivation mechanism is different in different cell types and that there is still some unknown VRAC subunit other than LRRC8. By culturing A240286S (A24) lung adenocarcinoma cells (A24) in an increasing concentration of cisplatin for a long time (for seven months) and then in an environment devoid of this compound, the (D-) A24cisPt8.0 cell line was obtained, which was resistant to cisplatin, oxaliplatin, and pemetrexed at a low level. Although cisplatin lowered the expression level of LRRC8D, it has been shown by the knockdown and overexpression experiments with LRRC8A and D that these proteins do not regulate the observed cisPt resistance [100]. VRAC currents were also present in the SNU-601 gastric cancer cell line independent of LRRC8A and almost completely absent in its cisplatin-resistant derivative SNU-601-R10 (R10). However, VRAC currents were found to be completely absent in SNU-601 cells, which were deficient in TTYH1 and TTYH2 and were restored by the expression of TTYH1 or TTYH2 [101]. This suggests that TTYH1 and TTYH2 may act as VRAC independent of LRRC8A.

## 3. CLC

### 3.1. Physiological Role of Clic Family of Chloride Channels

Chloride-conducting ion channels of the ClC family play an important role in most biological processes. They are polytopic membrane proteins and will form aqueous pathways through which anions are selectively allowed to pass down their concentration gradients. That is why the chloride ions flow between the cell, its compartment, and the outside is possible [102]. We can distinguish seven classes of ClC channels. If working properly, they will play a key role in the regulation of organelle volume and pH, factors that are important for the delivery of membrane transport proteins to the plasma membrane. The functions may vary significantly, e.g., CLIC1 functions range from ion homeostasis to cell volume regulation, transepithelial transport, and electrical excitability [103]. However, any mutation of the chloride channels genes might lead to various human diseases of muscle, kidney, bone, and brain, such as congenital myotonia, cystic fibrosis, osteopetrosis, epilepsy, or glioma [104].

The chloride channels ClC-3 might be found in both endosomes and synaptic vesicles and play a role in mechanisms of swelling of cells [99]. Most of them are not well studied; up to now, ClC-3 was found to be one of the causes of the drug resistance. All of the channels ClC-3 to ClC-7 will be present in endosomes. Among them, only ClC-5 and ClC-7 can reach the plasma membrane [105]. If all those channels are not working correctly, it might lead to numerous diseases, such as epilepsy, glioma, malaria or myotonia congenita. Defects of the chloride ClC-3 might lead to degeneration of hipocamp [106], ClC-5 creating the kidney stones [107] or chloride channels 7 may cause osteoporosis [108].

### 3.2. Role of ClC-3 Chloride Channels in Drug Resistance Phenomenon—Acidification Mechanism

Chloride channels are present in most of the cells. So far, what is interesting, is the fact that their overexpression is observed in numerous tumors. During the pass of time, questions if those channels might cause chemotherapy resistance arose. An experiment was performed in which Weylandt et al. [8] demonstrated that ClC-3 could participate in the acidification of the intracellular compartment. It was believed that it would promote the chelation of the etoposide in neuroendocrine tumor cells and, as a result, reduce the activity of this compound. The research used weak amine base acridine orange or the red fluorescent cyanine dye, DND-99/LysoTracker Red to stain live BON cells expressing ClC-3-GFP for intracellular acidic compartments. The chloride channels dyed with fluorescent compounds were found in the membrane of acidic vesicles. Briefly, changes in the acidity of intracellular organelles can be detected by determining the change in slope between the intensity of AO fluorescence emission in the far red (FL-3 channel) and the concentration of [AO]ext to which the cells are exposed [8]. It is established that intracellular acidic compartments may sequester basic anticancer drugs. They would typically accumulate passively in response to the pH gradient and thus contribute to drug resistance. As said earlier, the whole family of ClC channels has an enormous significance in the flow of ion currents between endosomes and the cell, including lysosomes. Thus it is possible to modify the pH of the whole cell and induce the drug resistance [109].

Cellular detoxification mechanisms in neoplastic cells are based on proton pumps and transporters and consequently the H^+^ efflux of cancer cells, resulting in a reversal of the average pH gradient. Drugs are sequestered and inactivated in intracellular acidic compartments. Afterward, they would dissipate their contents into the tumoral environment and thus contribute to the hyperacidified extracellular microenvironment of malignancy [110]. What is more, acidification is caused by overexpression of the ClC-3 channels and the Warburg effect. In cancerous cells, cellular energy metabolism changes from oxidative phosphorylation towards anaerobic glycolysis even in the presence of oxygen [78,111]. High rate of glycolysis results in extruding protons into the extracellular tissue, thus increasing its acidity [112]. It is a well-known fact that neutral substances can penetrate cell membranes better than positively or negatively charged ions [113]. According to pH-partition theory, at lower pH basic drugs, e.g., doxorubicin, undergo ionization. As a result, they would not easily penetrate the cell membrane, and their efficacy will be lowered. This is called the “ion trapping” phenomenon. The same problem usually occurs with other fundamental compounds such as anthracyclines, anthraquinones, and Vinca alkaloids. Weakly basic drugs will also tend to accumulate in lysosomes and endosomes that have an acidic lumen [114].

**Figure 4:**
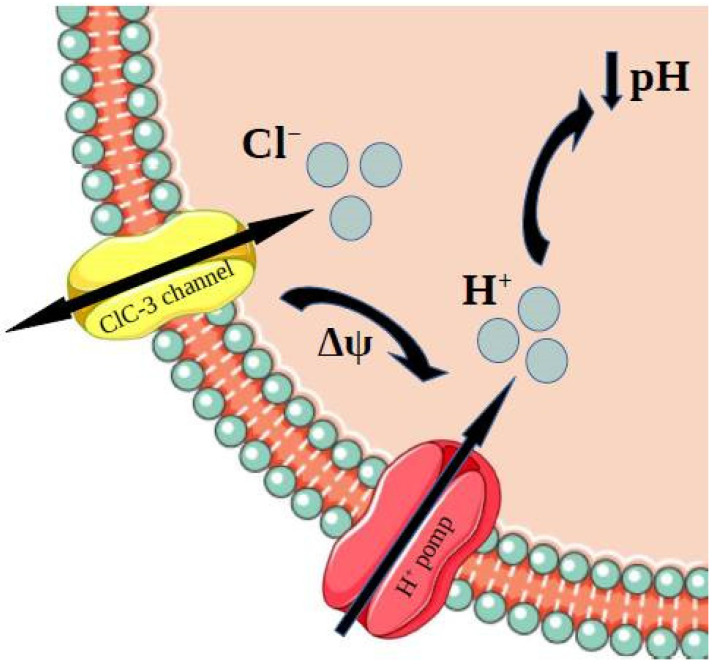
Cl^−^ channels, which are found mainly in intracellular compartments, have a distinct role in maintaining pH of these compartments. The reason for this is the opening of Cl^−^ channels, which provides the Cl^−^ influx. The transport of the chloride ions and the current caused by it enables the H^+^-ATP pump to work properly and, as a result, induces acidification [8,116].

Several studies have indicated an involvement of drug resistance and intravesicular acidity by comparing drug-sensitive and drug-resistant cell lines [115]. It might be assumed that it is the proton pump responsible for the acidification of the cell. However, it was found that the chloride channels also play an important role. Cl^−^ channels, which are found mainly in intracellular compartments, have a distinct role in maintaining the pH of these compartments. The reason for this is the opening of Cl^−^ channels, which provides the Cl^−^ influx. The transport of the chloride ions and the current caused by it allows the membrane potential to change. It is required for the acidification by H^+^-ATPase, and it enables the H^+^-ATP pump to work properly [8,116] (Figure 4). We should also mention the fact that numerous studies have demonstrated a need for low pH for the insertion of transport proteins into the plasma membrane and the cellular uptake of hormones and vitamins.

### 3.3. Role of ClC-3 Chloride Channels in Resistance to Cisplatin

Ye Xu et al. conducted another study in which they wanted to establish a connection between 5-nitro-2-(3-phenylpropylamino) benzoic acid (NPPB)-induced drug resistance to cisplatin, ClC-3 expression, and efficacy of cisplatin itself. In the research, there were used erythroleukemia K562 and RK562 cell lines. It was found out that the chloride channel blocker NPPB upregulated ClC-3, which made K562 and RK562 cells effectively avoid mitochondrion-mediated apoptosis [117]. As mentioned earlier, cisplatin (or cisplatinum, cis-diamminedichloroplatinum (II)) is a well-known drug used in cancer treatment. Its action is based on its ability to crosslink with the purine bases on the DNA, and thus interfering with DNA repair mechanisms, causing DNA damage, and subsequently inducing apoptosis in cancer cells [118]. The study by Yu Xe et al. presents evidence for the involvement of Cl^−^ channels in drug resistance—upregulation of the CIC-3 expression promotes acidification of intracellular compartments and induces sequestration of cisplatin, which consequently leads to cells avoiding cisplatin-induced apoptosis [117].

This knowledge might lead to looking for new therapeutic targets to improve the efficacy of anticancer drugs, including cisplatin. The inhibitors of v-ATPase such as bafilomycin A1 or lysosomotropic agents such as chloroquine might interrupt lysosome acidification and consequently suppresses autophagy by inhibiting autophagosome-lysosome fusion [119]. Studies carried out by Su et al. showed that silencing the ClC-3 resulted in inhibition of autophagy and Akt/mTOR pathway, and thus significantly enhancing apoptotic cells induced by cisplatin. What is more, their studies suggest that ClC-3 plays double roles in cisplatin-resistant mechanisms in U251 cells. Those chloride channels promote the Akt/mTOR pathway (by generating ROS by Nox), and ClC-3 induces acidification of acidic intracellular compartments such as late-endosome, lysosome, and mature autophagosome [120]. Although numerous studies are researching the role of ClC-3 in the MDR mechanism, it still needs further investigation.

### 3.4. Role of ClC-3 Chloride Channels in Drug Resistance Phenomenon—P-Glycoprotein Upregulation Mechanism

**Figure 5:**
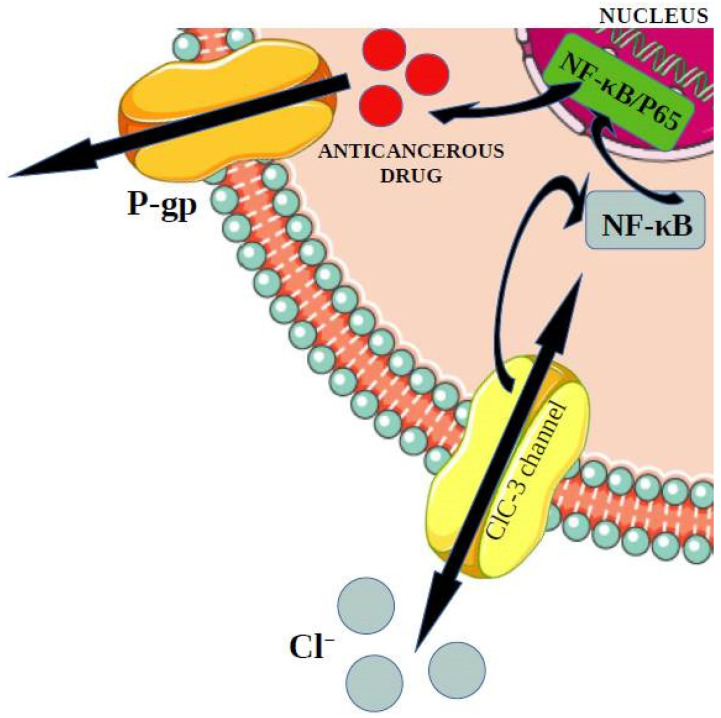
The increase in ClC-3 expression might activate the NF-κB. The activation of this factor can lead to the expression of MDR and P-glycoprotein. P-glycoprotein is one type of ATP-binding cassette. The transporter essential in the multi-drug resistance will bound with the ATP and get rid of the medicines out of the cell [122,123,125].

Overexpression of chloride channels ClC-3 genes and consequently increase of their activity, might lead to the development of breast cancer [121]. What is more, a change in the expression of those genes can also lead to creating multi-drug resistance. It was found that MDR might appear in previously sensitive cells MCF-7 (breast cancer cells lines) and A549 (P-glycoprotein lung adenocarcinoma cell lines). MDR is one of the most important causes of why chemotherapy is not efficient in treating cancer [122]. The change in expression of ClC-3 genes led to creating the multi-drug resistance MDR among previously sensitive cells. The research on mice showed that an increase in ClC-3 expression might activate the NF-κB [123]. According to growing evidence, it is worth mentioning that NF-kB (nuclear factor kB) participates in the regulation of immune responses and inflammation and might support a significant role in oncogenesis. NF-kB will regulate the expression of genes involved in many processes playing a significant role in the development and progression of cancer, such as proliferation, migration, and apoptosis [124]. The activation of this factor can lead to the expression of MDR and P-glycoprotein [123]. P-glycoprotein is one type of ATP-binding cassettes [122]. The transporter essential in the multi-drug resistance will bound with the ATP and participate in getting rid of the medicines out of the cell [125]. In this case, the P-gp pumps substrates such as doxorubicin (DOX) and paclitaxel (PTX) out of cancer cells, and as a result, it will cause a decrease in the accumulation of drugs inside tumor cells [126] (Figure 5).

To investigate the correlation between ClC-3 and P-gp, Qi Chen et al. carried out research in which they observed the effects of expression changes of ClC-3 or P-gp on the expression of P-gp or ClC-3, respectively. It was shown that both A549 and MCF-7 cells over-expressing ClC-3 showed significant upregulation of P-glycoprotein. Furthermore, they transfected si-ClC-3 causing high P-gp expression, which led to silencing ClC-3 expression in both A549/Taxol and MCF-7/DOX cells. It resulted in significant down-regulation of P-gp expression. On the other hand, overexpression of ClC-3 in drug-sensitive A549 or MCF-7 cells markedly upregulated P-gp expression and resulted in drug resistance. Spontaneous mammary cancer with ClC-3 overexpression in MMTV-PyMT/CLCN3 double transgenic female mice used in this research showed drug resistance to PTX and DOX that led to a significant decrease in survival times [123].

On the other hand, numerous researches indicate no link between expression of the ClC-3 genes and expression of the ATP-binding cassettes present in MDR. Both cells form the BON line, and those characterized by overexpression of the ClC-3 did not show the expression of the P-glycoprotein. Data presented by Weylandt et al. [8] study cannot correlate the level of ClC-3 expression and etoposide resistance because pH cannot be lowered beyond a certain threshold by increased ClC-3 expression [8].

### 3.5. Correlation between Clic-1 Chloride Channels Expression and Metformin Efficacy

Metformin is well-known medicine for treating type 2 diabetes. However, it was discovered that it could also perform anti-neoplastic impact by helping in decreasing the proliferation of cells and inducing apoptosis. Some research implies that the inhibition of the chloride channels CLIC-1 and thus inappropriate flow of the ions might be the cause of the observed metformin treatment effects [127]. On the other hand, newer researches show a link between the level of chloride channels CLIC-1 and the effectiveness of the medicine. Increased levels of those channels in cancerous cells were observed, and thus it became a tumor marker [128]. However, what is interesting is that this overexpression may strengthen the effectiveness of metformin. It was found that in gallbladder carcinoma cells, there is an overexpression of CLIC-1 compared to the noncancerous tissue. It is believed that metformin will silence the CLIC-1 genes and as a consequence, induce apoptosis and inhibit the proliferation of cancerous cells. This is possible why an increased amount of those chloride channels enable the metformin to work, yet it needs to be researched [129].

### 3.6. Mechanisms of Influencing Biochemical and Nuclear Paths by CLIC Family

It is still researched if Cl^−^ channels positively or negatively regulate the PI3K/AKT/mammalian target of rapamycin (mTOR) and signal transducer and activator of transcription (STAT)3 signaling pathways [130]. Liu et al. showed that ClC-3 activators with potent antitumor activities inhibited the PI3K/AKT/mTOR signaling pathway135. On the other hand, Wong et al. showed that the Cl^−^ channel inhibitor exerted suppressive effects on the PI3K/AKT and JAK/STAT3 signaling pathways [35]. Additional studies are needed to clarify the exact mechanisms and possibilities, as HER2 is an attractive therapeutic target for gastric, colorectal, and ovarian cancer, and the STAT3 signaling pathway is connected with HER2 overexpression in ovarian cancer [131]. Fujimoto et al. [130] carried out a study in which they showed that the siRNA-mediated inhibition of ClC-3 and ANO1 resulted in increased AKT phosphorylation and decreased STAT3 phosphorylation in MDA-MB-453 and YMB-1 cells, respectively. It is worth to mention that although there was observed an expression of the intracellular Cl^−^ channel protein CLIC1 in both cells, its siRNA-mediated inhibition did not elicit the transcriptional repression of HER2 [130].

However, Wu J. and Wang D. showed a role of CLIC1 in inducing drug resistance in human choriocarcinoma by positive regulation of MRP1. The study established JeG3 line cells. Knockdown of CLIC1 by shRNAs significantly increased cell sensitivity to MTX in JeG3/MTX cells or FUDR in JeG3/FUDR cells in vitro and in vivo [10]. Cell viability in response to drug treatment decreased by up to 60–75% after CLIC1 knockdown. CLIC1 functions by positive regulation of MRP1, which reduces the drug accumulation by using ABC transporters [125]. MRP1 will induce resistance to numerous anticancer drugs such as doxorubicin, MTX, VP, daunorubicin, and vincristine [132]. The Wu J. and Wang D. study found that knockdown of MRP1 significantly abolished CLIC1-mediated acquirement of chemoresistance in JeG3 cells. We might presume that CLIC1 exerts its chemoresistant property by promoting the expression of MRP1 in choriocarcinoma. However, no studies are fully explaining the molecular mechanism for the induction of MRP1 [10].

As we know, the tumor microenvironment plays an important role in the development and progression of tumors, and tumor-derived extracellular vesicles (EVs) are emerging as a pivotal part of the TME in human tumors [133]. That is why, studies on the exosome, which might transport both substances and tumor cells night clarify more mechanisms of drug resistance. Zhao K. Et al. carried out a study, in which they showed the role of CLCI1 in the development of resistance to vincristine in gastric cancer (GC). They established that the expression level of P-GP and Bcl-2 was consistent with the level of CLIC1 as a result of testing the expression of P-GP, Bcl-2, and Fas in GC cell line SGC-7901 after co-culturing with exosomes from SGC-7901/VCR [134]. However, in vivo study would be helpful to confirm the results in the future, as they studied the cells only in vitro. P-GP and its influence on drug resistance have already been discussed in this writing, for we will explain the Bcl-2 role in drug resistance. Bcl-2 is well-known for being a key regulator of the progression of apoptosis and MDR in multiple types of cancer [135]. The proto-oncogene bcl-2 appears to serve a critical antiapoptotic function, both in developing cancer and creating drug resistance [136].

### 3.7. Relationship between ClC5 and Bortezomib Resistance in Myeloma Cells

Multiple myeloma, a frequent malignancy of plasma cells, is treated by numerous anticancer drugs, including bortezomib (BZ). Bortezomib will inhibit the proliferation of myeloma cells, induce apoptosis, reverse drug resistance and block cytokine circuits, cell adhesion, and angiogenesis in vivo [137]. However, chemotherapy resistance is a major problem [138]. Zhang H. et al. explored the relationship between ClC5 and BZ resistance in multiple myeloma cells. It was found out that knockdown of ClC5 enhanced cells sensitivity to BZ in multiple myeloma cells, while ClC5 upregulation inhibited the BZ-induced decrease in cell viability. BZ treatment-induced autophagy and inhibition of autophagy augmented the cytotoxic activity of BZ against multiple myeloma cells. The reason for it might be ClC5 inducing autophagy by dephosphorylation, for inactivating the AKT–mTOR signaling pathway. That is because phosphorylation of AKT can activate the serine/threonine kinase mTOR, which will result in upregulating the autophagy [139].

## 4. Conclusions

Channels play an essential role in the multidrug resistance phenomenon, especially the ClC family and VRAC/VSOR. These are the chloride channels that researchers mainly focus on, as they could be the main hope for therapeutic targets. VRAC/VSOR channels are important cellular elements associated with the proliferation, migration, and multidrug resistance of cancer cells. Moreover, they appear to influence the MDR phenomenon in two ways (Figure 2). They are channels through which, thanks to the appropriate diameter concerning small-molecule medicinal substances [90,91], anti-cancer drugs such as cisplatin or carboplatin can enter the cell interior [26]. On the other hand, VRAC constituent proteins mediate signaling pathways that modulate drug-induced cell death [27] and facilitate AVD-related apoptosis induced by anti-cancer drugs such as cisplatin [117,120]. In the context of research on the role of VRAC, the identification of LRRC8 homologs as their components was crucial [22]. This made it possible to break the stagnation in research on the role of VRAC/VSOR. LRRC8A was determined to be a key subunit for VRAC/VSOR functioning that connects to a second LRRC8B-E subunit to form a fully functional channel. Its diameter depends on the type of the second subunit and, consequently, substances that can pass through the channel, including anti-cancer drugs such as cisplatin [22,23,26]. Still, however, there are many issues to be settled. Cisplatin-resistant cells with reduced VRAC activity have been described that do not show decreased expression of LRRC8 subunits relative to the cisplatin-sensitive parental cell line [95,96]. This leads to the hypothesis that other subunits can form VSOR/VRAC channels. Recently, the TTYH1 and TTYH2 subunits have been identified as novel components of VRAC in some cancer cells [101]. Therefore, further studies on various types of neoplastic cells are necessary to identify other proteins that can form functional VRAC/VSOR channels and elements that may affect the functioning of the channels. Furthermore, the molecular mechanisms by which VRAC/VSOR influences proliferation, migration, apoptosis and cisplatin resistance remain to be clarified.

Furthermore, the second ClC family—also known as CLIC channels—plays an important role in most biological processes by selectively allowing anions to pass down their concentration gradients, thus creating the ions flow between cells, its compartment and the outside is possible. Their overexpression might be found in numerous tumors, such as breast cancer. What is more, it turned out that it plays an important role in creating drug resistance [102]. The best-researched chloride channel is ClC-3 and, both, its acidification and (NPPB)-induced drug resistance mechanisms [8,117]. Change of the membrane potential caused by chloride ions current leads to the acidification by H^+^-ATPase (enabling the H^+^-ATP pump to work appropriately) [8,116]. However, further research considering whether there is a link between the expression of the ClC-3 genes and the expression of the ATP-binding cassettes present in MDR in the nuclear path mechanism is needed. There might be found evidence for both present correlation and no link at all [8]. Despite a smaller amount of research, CLIC1 might induce drug resistance in human choriocarcinoma by positive regulation of MRP1 [10]. Furthermore, ClC5 is believed to induce autophagy and, as a consequence, cause resistance to bortezomib (an anticancer drug) in multiple myeloma cells [9]. Further research is needed to discover all of the mechanisms and chloride channels taking part in the drug resistance phenomenon and thus create new, more efficient treatment possibilities.

## Data Availability

Not applicable.
